# Transforming Cancer Care: Genomic Profiling, AI, and Next-Generation Drug Delivery Systems

**DOI:** 10.2174/0113892029406752251110100414

**Published:** 2026-02-24

**Authors:** Kaushal Aggarwal, Priya Jindal, AkashVikal, Preeti Patel, Balak Das Kurmi

**Affiliations:** 1 Department of Pharmaceutics, ISF College of Pharmacy, GT Road, Moga-142001, Punjab, India;; 2 Department of Pharmaceutical Quality Assurance, ISF College of Pharmacy, GT Road, Moga-142001, Punjab, India;; 3 Department of Pharmaceutics, ISF College of Pharmacy and Research, Rattian Road, Moga-142001, Punjab, India;; 4 Department of Pharmaceutical Chemistry, ISF College of Pharmacy, GT Road, Moga-142001, Punjab, India

**Keywords:** Cancer genomics, immunogenomics, liquid biopsy, oncology, CRISPR

## Abstract

Cancer genetics plays a revolutionary role in the field of oncology by providing novel insights into tumor evolution, heterogeneity, and treatment targets. Recent advances in sequencing technology, such as whole-genome and transcriptome sequencing, have enabled the precise molecular profiling of cancers, leading to the development of targeted and individualized treatment approaches. Liquid biopsy has emerged as a non-invasive approach for collecting samples, utilizing blood or other body fluids to identify tumor cells, molecular changes, and metabolites. Liquid biopsy helps to detect various biological markers, including ctDNA, CTCs, and exosomes. Immunogenomics has revolutionized cancer management through the use of immune checkpoint inhibitors and personalized neoantigen vaccines. Furthermore, the integration of machine learning facilitates the examination of extensive genomic datasets, enabling the detection of patterns, the prediction of treatment responses, and the identification of novel therapeutic targets. Innovative drug delivery technologies, such as nanoparticle-based and CRISPR-mediated genome editing, provide promise for more effective and less harmful treatments. This review discusses recent advancements in cancer genomics, including liquid biopsy, tumor heterogeneity, immunogenomics, and the function of machine learning in the analysis of genomic data. By understanding ethical and social issues, successful implementation can be possible. The combination of genomics, immunogenomics, and liquid biopsy holds promise for novel, personalized cancer treatments, indicating a new era in cancer drug development.

## 1 INTRODUCTION

**Fig. (1):**
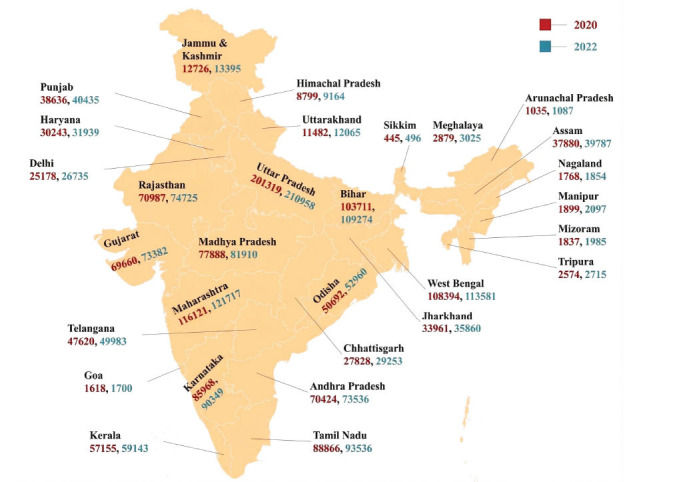
Cancer cases as per the Government of India, Ministry of Health and Family Welfare, Department of Health and Family Welfare, in the years 2020 and 2022.

**Fig. (2):**
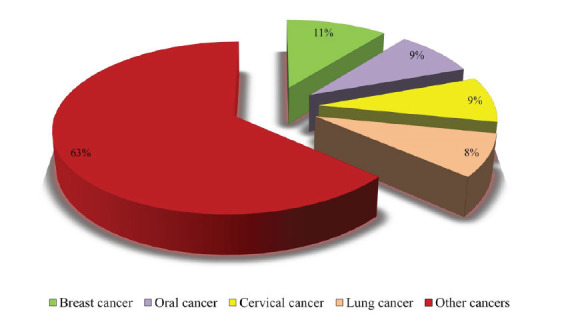
Mortality rate due to cancer as per the WHO in India in the year 2022.

Cancer is a genomic disorder where inherited germline aberrations or acquired epigenetic changes contribute to the persistence and proliferation of cancerous cells. According to the WHO, cancer is characterized by the rapid formation of abnormal cells that expand beyond their normal limits, potentially invading nearby tissues and spreading to other organs [1]. As per the Indian Council of Medical Research-National Cancer Registry Programme [2], the estimated number of cancer cases in India in the years 2020 and 2022 are shown in Fig. **1**, and the estimated number of mortality cases due to cancer in India (2022) as per WHO is depicted in Fig. **2**. Advancements in molecular diagnostic tests and the development of effective therapies have improved the identification and targeting of genomic abnormalities, including small insertions and deletions, single-nucleotide variants, and structural modifications. Complex biomarkers, such as Microsatellite Instability (MSI), homologous recombination deficiency, and Tumor Mutational Burden (TMB), play a crucial role in cancer diagnosis and treatment. With the growing number of clinically actionable and pan-cancer biomarkers, along with the increasing accessibility of sequencing technology, comprehensive molecular approaches such as Whole-Transcriptome Sequencing (WTS), collectively known as Whole-Genome Transcriptome Sequencing (WGTS), and Whole-Genome Sequencing (WGS), are being progressively incorporated into cancer diagnostics. Recent studies further validate the adoption of WGS and WGTS, supporting their potential to become a standard in routine oncology care [3-5]. The identification of DNA as the molecular basis of inheritance and the elucidation of its structure strengthened the link between genetics and cancer. This connection was further supported by demonstrating that agents causing DNA damage and mutations also induce cancer. This historical context underscores the crucial role of the genome in cancer development [6-8]. These discoveries have facilitated the development of highly specific and effective therapies, making certain hematologic malignancies manageable and curable. Despite the initial limitations of sequencing technology, efforts to identify cancer-specific mutations began with the use of high-throughput Polymerase Chain Reaction (PCR) amplifications, followed by sequencing of well-established cancer genes [9, 10]. Nowadays, pharmaceutical companies are conducting various clinical trials of drugs, targeting specific proteins thought to drive oncogenesis, to significantly reduce tumor burden in some advanced metastatic patients (Table **1**). By identifying specific genetic alterations, known as oncogenic drivers, which act as a source for uncontrolled cell growth, researchers have developed targeted therapies for distinct subgroups of Non-Small Cell Lung Cancer (NSCLC) patients [11, 12].

We conducted a comprehensive literature search using electronic databases including PubMed, Scopus, Web of Science, and Google Scholar. The search included publications from January 2012 to June 2025, using keywords such as *genomic profiling in cancer, next-generation drug delivery systems, personalized cancer therapy,* and *nanotechnology in cancer treatment.* We have included peer-reviewed articles, systematic reviews, and relevant clinical trials that addressed advancements in genomics and drug delivery specific to cancer care.

However, differential patient responses prompted efforts to correlate specific mutations with treatment response, leading to the identification of mutations associated with response to targeted therapy in non-small cell lung adenocarcinomas. These advancements have significantly improved survival rates for certain patient groups, pushing median survival beyond two years. Many techniques exist to identify alterations in the cancer genome, epigenome, transcriptome, and proteome. Genomic technologies like Whole-Exome Sequencing (WES), WGS, gene panels, hotspot testing, and histopathology reveal distinct features of cancer [19-21]. Transformative sequencing technologies, such as massively parallel sequencing (MPS), have emerged, enabling the unbiased sequencing of tumor and normal genomes and the identification of somatic mutations [22]. These technologies have enabled large-scale identification of somatic alterations across various cancer genomes, providing insights into the diverse mechanisms by which cancer genomes evolve. Support Vector Machine (SVM), Convolutional Neural Network, language model, Transformer-based language model, and large-scale language model are some applications of AI techniques in genetics, which are one of the highly productive research fields [23]. Challenges in solid tissue malignancies, such as the difficulty of obtaining post-treatment biopsies, have limited studies on changes to the cancer genome. Despite these challenges, a fundamental understanding of the tumor genome landscape has been established for common tumor types, and the clinical application of genomics is now a clear next step. Major programs such as The Cancer Atlas (TCGA) and the Cancer Genome Consortium (ICGC) have been pivotal in identifying key mutations that give cancer cells a growth advantage and could serve as potential therapeutic targets [24, 25]. This review discusses recent advancements in cancer genomics, including tumor heterogeneity, liquid biopsy, immunogenomics, and the role of Machine Learning (ML) in genomic data analysis.

## 2 TUMOR EVOLUTION AND GENOMIC HETEROGENEITY

With the understanding of tumor evolution and genomic heterogeneity, researchers may develop effective cancer therapies. Intra-tumor heterogeneity, which refers to the presence of different genetic and epigenetic subpopulations within a tumor, promotes tumor evolution and presents a significant challenge in cancer treatment (Table **2**).

**Fig. (3):**
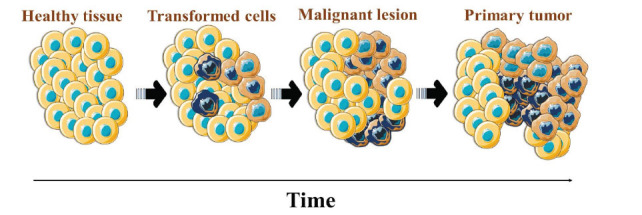
The schematic representation demonstrates the stepwise progression from healthy tissue to a primary tumor, including the transformation of cells and the development of malignant lesions. The different colors of cells illustrate the heterogeneity within the same subpopulation of cancer cells.

Genomic instability, a major cause of genetic heterogeneity in cancer, increases the mutation rate and influences the evolution of the cancer genome (Fig. **3**) [40]. Tumor subclones, exhibiting differential gene expression due to genetic and epigenetic heterogeneity, contribute to the complexity of tumor evolution [41]. Cancer cells continuously adapt to their surrounding environment, allowing them to resist treatment and suppress the human immune system's ability to recognize and eliminate these cells. In distinct forms of cancer, which vary in their genetic and epigenetic heterogeneity, a similar evolutionary pattern emerges for a given tumor. The interaction between genetic and epigenetic factors results in complex tumor evolution, affecting the treatment response as well as disease progression [42]. To develop targeted and personalized treatment approaches, a thorough understanding of the evolutionary dynamics of genetic heterogeneity is essential [43]. By unraveling the evolutionary dynamics of tumors, researchers have focused on developing targeted therapies that account for the diverse genetic and epigenetic profiles of individual tumors. Such personalized approaches hold promise for enhancing the treatment outcomes of cancer patients. However, tumor evolution and genomic heterogeneity are complex processes that significantly impact cancer progression and treatment. The evolutionary dynamics of cancer cells, driven by genetics (dysregulation of oncogenes and tumor suppressor gene) and environmental factors, underscore the complexity of tumor evolution [44]. This deeper understanding of tumor evolution holds the potential to transform the landscape of cancer care, offering new hope for improved patient outcomes and survival rates.

## 3 LIQUID BIOPSY

Liquid biopsy is a non-invasive method for collecting samples from blood or other body fluids to detect molecular changes, tumor cells, and metabolites [45, 46]. In asymptomatic individuals, it is invaluable for early cancer detection and intervention. Compared to tissue biopsies, liquid biopsy plays an important role in early screening. Blood and urine are the most commonly used specimens. Since liquid biopsy is minimally invasive and easier to perform, it offers the potential for continuous monitoring of tumor progression [47, 48]. Various molecular markers can be detected through liquid biopsy, including circulating tumor DNA (ctDNA), Circulating Tumor Cells (CTCs), Tumor-Educated Platelets (TEPs), Tumor-derived Extracellular Vesicles (TEVs), and circulating free RNA (cfRNA) [49]. Current research primarily focuses on detecting exosomes, ctDNA, and CTCs.

### 3.1 Circulating Tumor Cells

**Fig. (4):**
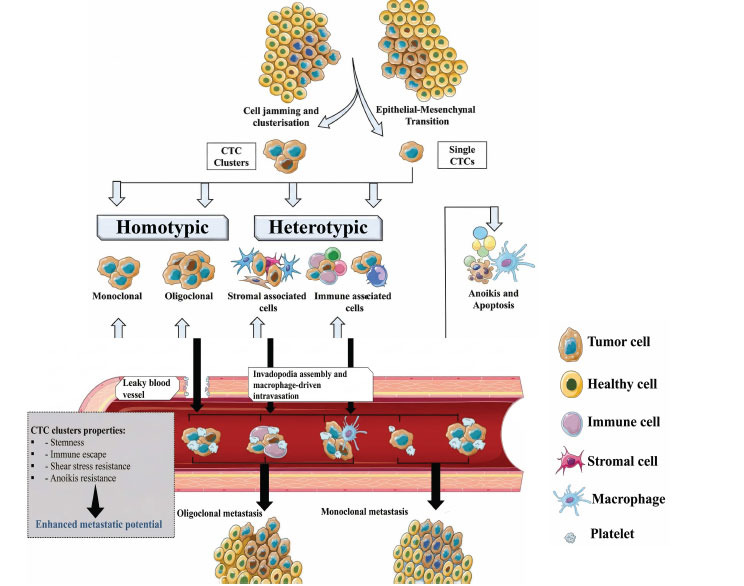
A schematic illustration describes the process of tumor cell dissemination, where tumor cells detach from the primary tumor site, enter the bloodstream or lymphatic vessels, circulate through the body, and ultimately colonize secondary sites, leading to the formation of metastatic tumors.

CTCs are cells shed from primary and metastatic tumors into the bloodstream or lymphatic vessels of cancer patients, circulating in peripheral blood [50]. Although their proportion in blood is low (approximately one CTC per million leukocytes), most CTCs do not survive beyond 1 to 2.5 hours [48, 51]. Despite this, CTCs hold promise as a valuable tool for cancer diagnosis, aiding clinical decision-making and research (Fig. **4**) [52]. Despite this, CTCs hold promise as a valuable tool for cancer diagnosis, aiding clinical decision-making and research. Detection methods rely on specific marker expressions, including epithelial cell adhesion molecule (EpCAM), vimentin, and N-cadherin, using techniques such as EpCAM enrichment, immunomagnetic separation, and microfluidic devices [53]. Among these, the CellSearch^®^ method remains the only FDA-approved technique for monitoring CTC levels in blood samples [54].

### 3.2 Exosomes

**Fig. (5):**
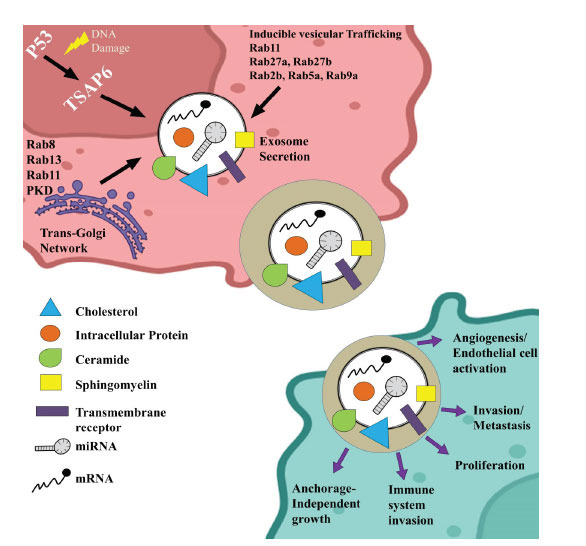
Schematic representation of exosomes secretion in a cancer cell shows exosomes being generated and fused with the plasma membrane, released through an integral pathway involving the trans-Golgi network. The secreted exosomes can be taken up by neighboring cells, potentially inducing cancer progression. Reproduced from: Recent advances in liquid biopsy technologies for cancer biomarker detection, Sensors & Diagnostics, DOI: https://doi.org/10.1039/D2SD00010E.

Exosomes are considered miniature versions of their parental cells due to their complex composition, which includes uniquely sorted proteins, lipids, nucleic acids, and other components [55]. Secreted by nearly every cell type, exosomes are characterized by their endosomal origin and small size, typically ranging from 30 to 100 nm in diameter. These exosomes can be released through the trans-Golgi network or *via* inducible release (Fig. **5**) [56]. The trans-Golgi network serves as a primary sorting station for secretory pathways, processing extracellular materials and recycled molecules from endocytic compartments before directing them to various subcellular destinations. Additionally, proteins from the Rab family of small GTPases are crucial for regulating intracellular vesicular trafficking, including cytoskeletal transport, vesicle budding, and docking/fusion activities [57].

### 3.3 Circulating Tumor DNA

ctDNA is a type of extracellular nucleic acid found in circulation [58]. It can be extracted from the bloodstream and is derived from the tumor. However, employing ctDNA sequencing for cancer screening encounters substantial challenges [59, 60]. In asymptomatic individuals, the concentration of ctDNA is relatively low, ranging between 1 and 15 ng/mL. To achieve optimal sensitivity, especially in breast cancer screening, large blood samples, typically between 150 and 300 mL, are needed [61]. Currently, ctDNA assays exhibit higher efficacy compared to traditional cancer-derived antigens, such as prostate-specific antigens and carcinoembryonic antigens [62]. Several studies support the potential use of ctDNA for early lung cancer diagnosis, detecting mutations like Kirsten Rat Sarcoma Virus (KRAS) and *TP53* as much as 2 years before cancer diagnosis. Beyond DNA mutations, combining ctDNA level quantification and DNA methylation enhances the robustness of results. Notably, the detection of SEPT9 gene methylation is FDA-approved for colorectal cancer screening, surpassing protein markers in terms of sensitivity and specificity [63-65].

In the realm of treatment selection and prognosis, ctDNA sequencing provides a real-time snapshot of tumor-specific molecular profiles, guiding precision medicine [66]. Its short half-life allows for dynamic monitoring of treatment effects, aiding in treatment adjustment and prognosis assessment [67]. Regulatory bodies have approved ctDNA-based *EGFR* mutation testing for therapy guidance in NSCLC patients [68, 69]. For those undergoing immunotherapies, ctDNA emerges as an early marker of therapeutic efficacy, predicting survival outcomes more accurately than conventional methods. The most commonly used technologies for mutation detection in ctDNA are Scorpion Amplified Refractory Mutation System (ARMS) PCR, droplet digital PCR (ddPCR), and NGS [70].

**Fig. (6):**
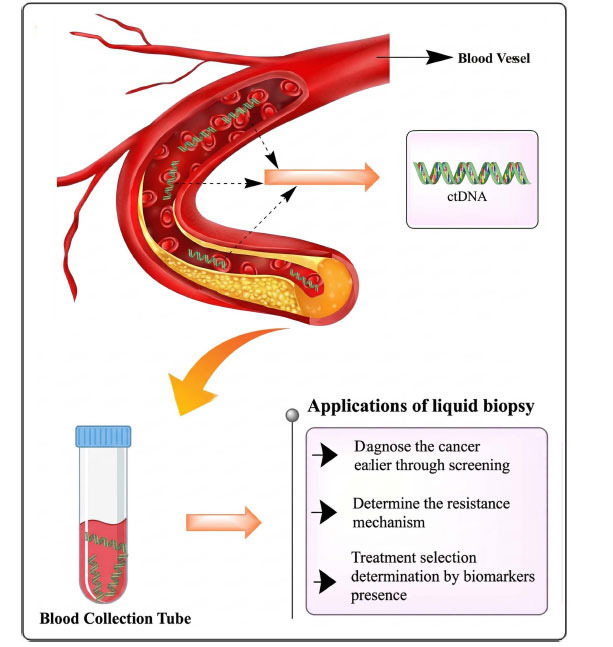
The figure illustrates the use of ctDNA in liquid biopsy. ctDNA released into the bloodstream is collected through a blood sample and analyzed.

Despite these advancements, ctDNA biological challenges include its highly fragmented nature and low concentration in the blood. Isolating ctDNA for quantification is challenging due to the potential loss or degradation of small fragments. Biological knowledge gaps, such as clearance rates and the quantitative relationships between circulating tumor DNA (ctDNA) and early cancer development, hinder further clinical applications (Fig. **6**) [71]. ctDNA also faces challenges in identifying low-frequency mutations due to limited sensitivity. To overcome this, Wang *et al.* (2024) present ‘HiCASE,’ a highly sensitive and specific method to detect ctDNA mutations, utilizing a PCR-based CRISPR approach coupled with restriction enzymes [72]. Kamps *et al.* (2017) described the use of NGS in liquid biopsies across a range of cancers, including lung, HCC, gynecologic, pediatric, gastrointestinal tract tumors, and urothelial cancers [73]. The NGS-based analysis of liquid biopsy samples does not require an invasive biopsy of the tumor tissue and provides significant advantages in early cancer detection. It also supports monitoring of treatment response, identification of potential therapy resistance, and helps guide decisions for alternative treatment options (Table **3**) [74, 75].

## 4 IMMUNOGENOMICS

**Fig. (7):**
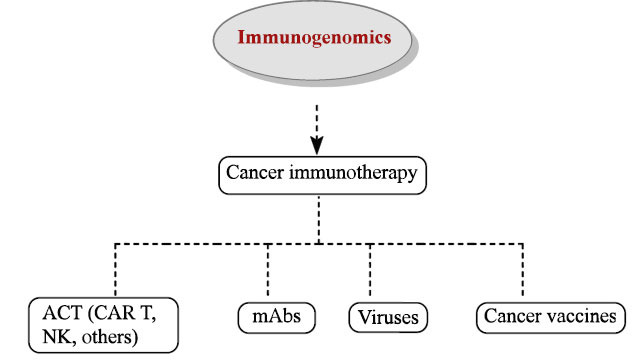
Potential of immunogenomics in cancer treatment.

Immunogenomics encompasses various genomics-based inquiries that delve into the interaction between the cancer and the immune system (Fig. **7**). Early work in melanoma highlighted the tumor's extensive immune interactions, paving the way for immunotherapies like checkpoint blockade therapy.

Researchers have developed computational techniques and ML algorithms to retrieve therapeutically valuable information from genomic and transcriptomic sequencing data from bulk tissue or single cells, enabling the exploration of tumors and their microenvironments [76]. These computational immunogenomic techniques can predict responses to cancer immunotherapies, such as Immune Checkpoint Inhibitors (ICIs), based on cancer-extrinsic and cancer-intrinsic factors that can be identified using sequencing, including neoantigens, TMB, and the presence of immune cells [77]. Neoantigens are novel antigens that arise from mutations and can also stimulate an immune response against tumors [78]. Genomic studies in melanoma revealed a high mutation rate, particularly due to UV damage, and identified potential biomarkers for immunotherapeutic responses. This groundwork proved pivotal in the development of revolutionary immunotherapies, such as checkpoint blockade therapy, which harnesses the immune response of the body to combat cancer [79]. Scientists have forged ahead, leveraging computational methodologies and ML algorithms to extract valuable clinical insights from extensive collections of genomic and transcriptomic sequencing data. These approaches are essential in evaluating both bulk tissue and individual cells, providing unparalleled insights into malignancies and their complex microenvironments [80]. Through computational immunogenomic assessment, researchers have been able to predict responses to cancer immunotherapies, like ICIs. These predictions are based on a comprehensive analysis of cancer-specific characteristics and extrinsic factors, identified using sequencing techniques, which include metrics such as TMB, neoantigens, and the presence or absence of diverse immune cells within the tumor environment [81].

Melanoma has emerged as a focus in genomic research due to its extensively high mutation rate, predominantly caused by UV-exposed DNA damage. These investigations have unveiled potential biomarkers that hold potential in predicting and understanding responses to immunotherapeutic interventions [82]. The recent progression in the field of immunogenomics continues to offer comprehensive insights into the intricacies of cancer-immune interactions, offering promising personalized and precise treatments based on genomic information that hold the potential to transform cancer care paradigms [83]. Clinical trials exploring immunotherapy in melanoma have demonstrated significant responses, leading to FDA approvals for anti-cytotoxic T lymphocyte antigen 4 (CTLA-4) and anti-PD-1 therapies [84]. These immunotherapies have expanded into clinical studies for other cancer types, such as bladder cancer and non-small cell lung cancer [85]. The connection between high mutation rates and immunotherapeutic responses has raised questions about whether mutational load alone predicts efficacy or if a more nuanced evaluation of neoantigens is necessary [86].

Ott *et al.* (2017) found that a personalized neoantigen vaccine is safe, feasible, and has the capacity to induce strong T-cell responses in clinical settings without being affected by prior or current therapies. This vaccine approach aims to address two significant challenges in cancer treatment, namely tumor heterogeneity and the selective targeting of tumors. The study observed the induction of de novo T-cell clones that can identify multiple patient-specific neoantigens, including autologous tumor cells and endogenously processed antigens. By targeting a wide range of malignant clones within each patient, the vaccine addresses tumor heterogeneity and reduces the risk of tumor escape due to antigen loss [87]. Future neoantigen vaccine trials will use improved methods for predicting antigen presentation to boost the proportion of neoantigens activating tumor-reactive T cells, as well as test their synergy with checkpoint inhibition and other immunotherapeutics [88].

However, mouse models and genomic studies have provided insights into the interaction between cancer and the immune system, identifying tumor-specific mutant antigens (neoantigens) [89]. Human studies have correlated high mutational loads with positive responses to checkpoint blockade therapies, raising questions about the predictive quality of mutational load compared to other markers like programmed death-ligand (PD-L1) and PD-1 protein expression [90]. These approaches, combined with the blockade of CTLA-4, have become pivotal in the era of immunogenomics, fundamentally altering the landscape of cancer immunotherapy. Tumor cells develop immune tolerance and evade detection by host CD4+ and CD8+ T cells within the tumor microenvironment, enabling them to escape immune monitoring [91]. While current genomic testing benefits only 10–15% of patients, improved cancer genome analyses have uncovered highly specific neoantigens from somatic mutations. These neoantigens and their corresponding T cells in the tumor environment play a crucial role in various cancer immunotherapies, such as ICIs [92]. Targeting patient-specific neoantigens holds immense promise for personalized cancer treatment. However, accurate prediction and selection of these neoantigens remain a challenge. Yet, precise identification could fast-track personalized immunotherapies, such as cancer vaccines and engineered T-cell therapy, offering tailored solutions for cancer patients [93]. This evolving field of immunogenomics leverages cancer genomics to design personalized vaccines based on tumor-specific neoantigens, offering a potential avenue for patients who have exhausted other treatment options (Table **4**).

## 5 MACHINE LEARNING IN GENOMIC DATA ANALYSIS

ML is a branch of artificial intelligence (AI) that involves enabling computers to make decisions from data without being directly programmed. It helps to process large amounts of complex data, identify intricate patterns, and develop predictive models that can guide personalized treatment and public health approaches [98]. It also plays an increasingly pivotal role in the analysis of extensive genomic datasets, offering the capability to discern patterns, predict treatment responses, and uncover novel therapeutic targets [99]. The goal of the ML approach is to acquire knowledge from historical or current data and use that understanding to make forecasts or decisions for unspecified future data metrics [100]. In genomic research, ML algorithms are widely applied across various domains. A well-established use case is genome annotation and predicting the effects of genetic variations, where ML techniques have become essential in bioinformatics processes [101]. These algorithms contribute significantly to enhancing our understanding of genomic variations and their potential functional consequences. The impact of ML extends into disease diagnosis and prognosis, where these algorithms adeptly analyze genomic data to identify patterns, enabling accurate disease diagnosis, prediction of disease progression, and informed treatment decisions [102].

Moreover, in liquid biopsy analysis, ML techniques prove valuable in scrutinizing data from various sources like CTCs and DNA, providing insights into cancer progression and treatment response [103]. ML techniques effectively identify disease-causing genomic variants by differentiating between pathogenic and benign variants. This significantly improves the accuracy of genetic diagnoses and enables more targeted treatment strategies for genetic disorders [104]. ML, particularly deep learning, contributes to improving gene editing tools like clustered regularly interspaced short palindromic repeats (CRISPR), enhancing precision in gene therapy strategies [105, 106]. Anticipating virus evolution, such as influenza and Severe Acute Respiratory Syndrome Coronavirus 2 (SARS-CoV-2), is made more effective with ML, aiding public health efforts to stay ahead of outbreaks and formulate timely interventions [107]. Furthermore, ML algorithms play a crucial role in variant calling, *i.e*., the computational process of detection and annotation, enhancing the characterization of genetic variants in genomic data. These techniques reveal intricate relationships between gene expression levels and disease outcomes, furnishing researchers with invaluable insights that may hold the keys to unlocking novel therapeutic targets [108, 109]. In the vast field of drug discovery and development, the synergy between machine learning and large datasets is transforming the landscape [110]. ML algorithms facilitate the identification of potential drug targets, streamline the optimization of drug candidates, and even predict clinical trial outcomes, ushering in a new realm of efficiency and efficacy in pharmaceutical research [111]. Due to this, the field of personalized medicine has been greatly impacted by the excellence of ML in analyzing genomic data. This analytical capability enables the development of customized treatment plans for cancer patients, harnessing the power of individual genetic profiles to predict treatment responses with high precision and efficacy [112]. This highlights the significant and growing impact of ML on genomic data analysis, as researchers continually refine algorithms to extract valuable insights from the complexity of genomic datasets [113].

### 5.1 Multi-Omics Integration in Cancer Research

The word “omics” refers to the study of molecules in a biological sample. The word “omics” encompasses a wide range of studies, including genomics, proteomics, metabolomics, transcriptomics, and others [114-116]. Nowadays, omics approaches have gained various novel advances in cancer research. In oncology studies, it is widely recognized that cancer involves unavoidable molecular changes at different levels of biological organization. Omics technologies have thus facilitated a deeper exploration of cancer-related processes, allowing scientists to unravel its unique molecular characteristics [117].

Genomics is one of the most basic levels among the omics branches. In the field of oncology, the approach is known as cancer genomics [118]. Cancer genomics is the study of genetic changes associated with cancer development and progression, which helps to promote personalized cancer therapy [119]. Since cancer is largely caused by changes and mutations at the genome level, which help identify cancer-specific molecular signatures and the underlying mechanisms, advances in genomic-level research and related technologies aid in cancer management, from detection to treatment [120]. In this regard, DNA sequencing methods, from the first generation to next-generation sequencing (NGS), have unlocked new avenues for understanding the genetic codes of organisms, advancing research in the field [121]. Large-scale initiatives, such as the Human Genome Project, HapMap, and genome-wide association analysis [122], can be seen as advances in human genome research. Additionally, in cancer research, worthwhile projects such as TCGA, ICGC, and the Cancer Genome Project have been developed by analyzing DNA sequences to focus on cancer assessment in individuals [118].

Significant advancements have been made since the discovery of genomics, particularly in the field of cancer research. One of the major advantages in this field has been the identification of oncogenes (such as the KRAS family proto-oncogenes [123], EGFR [124], and the phosphatidylinositol-3-kinase (PI3K)/AKT/mammalian target of rapamycin (mTOR) signaling pathway [125]). The precise identification of the role of these oncogenes in the carcinogenic process has led to their recognition as suitable drug targets for cancer treatment [125].

## 6 THERAPEUTIC DELIVERY IN GENOMIC CANCER CARE

It involves utilizing genetic information to design and administer treatment tailored to an individual's specific genetic makeup. This personalized approach aims to enhance treatment efficacy while minimizing adverse effects [126, 127]. Targeted therapy in cancer treatment involves using drugs or substances that specifically interfere with molecules crucial for cancer cell growth and spread. It focuses on identifying the unique characteristics of cancer cells and minimizing harm to healthy cells. Before treatment, molecular profiling of the tumor is conducted to pinpoint specific targets for therapy, such as proteins, receptors, or genetic mutations [10, 128]. Various types of targeted therapies, including small-molecule drugs and monoclonal antibodies, aim to disrupt these identified targets, such as Herceptin for HER2-positive breast cancer or imatinib for certain genetic mutations in leukemia [129].

Guo *et al. (*2019) developed a technique using antibodies and nanolipogels to deliver CRISPR-associated protein 9 (CRISPR/Cas9) plasmids directly into triple-negative breast cancer cells. By targeting the Lipocalin 2 gene, they achieved approximately 81% suppression efficiency, resulting in a 77% reduction in the growth rate of the tumor. This suggests that the tumor-targeted nanolipogel system acts as an efficient and precise delivery strategy for CRISPR-based genome editing customized for specific targets [130]. The delivery of modified genes to cancer cells comprises numerous methods that aim for precise and targeted transference of altered genetic material to target cells, such as viral vectors, non-viral vectors, electroporation, gene gun technology, CRISPR/Cas9 delivery systems, and injectable hydrogels. Viral vectors are used to transport therapeutic genes into cancer cells, such as adenoviruses, lentiviruses, and adeno-associated viruses, while non-viral vectors consist of synthetic carriers like liposomes, polymers, or nanoparticles that encapsulate and transport genetic payloads. Electroporation uses an electric field to produce temporary pores in the cell membrane, facilitating gene entry, whereas the gene gun method propels modified genetic material-coated particles into cells. The CRISPR/Cas9 system can be delivered *via* viral or non-viral vectors for precise genome editing within cancer cells. Injectable hydrogels consist of genetic materials that are directly administered for the controlled release of therapeutic genes specifically targeting cancer cells within the tumor microenvironment. These varied methodologies exhibit distinct advantages and limitations in terms of efficiency, specificity, safety, and scalability, warranting ongoing research and refinement for improved efficacy and reduced off-target effects in gene delivery to cancer cells. Drug delivery systems are pivotal in genomic cancer care, revolutionizing treatment precision by transporting therapeutic agents to specific cells or tissues based on genetic markers [130]. Nanoparticles, minute particles engineered typically within the range of 1 to 100 nm, offer targeted drug delivery by binding to receptors or proteins overexpressed in tumor cells, minimizing side effects while maximizing drug efficacy [131-134]. Liposomes, lipid-based vesicles, possess versatility in encapsulating diverse drugs and can be tailored to target specific cancer cells, ensuring biocompatibility and customizable sizes for optimal circulation and uptake [135]. Polymer-based systems, utilizing biodegradable polymers, allow for controlled drug release and targeted delivery, enabling customization for sustained therapeutic impact [136]. These systems hold promise in refining cancer treatments, yet their development necessitates stringent testing and personalized approaches to match individual genetic profiles and cancer characteristics for clinical application.

Liu *et al.* (2018) developed a dual-targeted delivery system using polymer and inorganic hybrid nanoparticles. It comprised a CRISPR/Cas9 plasmid targeting Cyclin-Dependent Kinase 11 (CDK11), which was encapsulated within the core of nanoparticles composed of protamine sulfate, calcium carbonate, calcium phosphate, and carboxymethyl chitosan. The S1411 aptamer ligands synergistically interact through electrostatic interactions to enhance binding [137]. Furthermore, combinatorial approaches in cancer therapy involve treatments based on a patient’s genetic profile to maximize effectiveness and minimize resistance within cancer cells, such as targeted therapy, immunotherapy, chemotherapy, or radiation [138]. By targeting multiple pathways simultaneously and potentially generating synergistic effects, these combinations aim to overcome resistance mechanisms and reduce the likelihood of cancer cells adapting to treatment [139]. Moreover, by using lower doses of individual drugs and selectively targeting tumor vulnerabilities, these approaches seek to minimize side effects on healthy tissues [140]. Continuous monitoring and dynamic adjustments to treatment plans based on tumor responses further optimize efficacy. However, challenges such as the complexity of treatment design, cost, and accessibility persist, necessitating ongoing research and clinical validation to fully harness the potential of these personalized combinations in cancer care [10]. The current genomic approaches are listed in Table **5**.

## 7 ETHICAL AND SOCIAL IMPLICATIONS

Cancer genomics introduces a multitude of ethical and social considerations that demand thoughtful examination. Ethical concerns within cancer genetics encompass the responsible use of genetic tests for high- and average-risk individuals, the genetic testing of children, the familial repercussions of genetic information, safeguarding genetic privacy, and the complexities posed by genetic research. Integrating genomics into clinical oncology raises additional issues regarding the privacy and confidentiality of genetic data, fair distribution of healthcare resources, and ensuring meaningful informed consent for genetic testing [147, 148]. Moreover, the ethical, legal, and social implications of cancer research are becoming increasingly intricate, demanding meticulous attention, especially when collecting biological samples and associated data for potential genetic or genomic studies. Federal regulations mandate ethical practices in the collection of research specimens and data to protect the rights and welfare of biospecimen donors and ensure the fair and just conduct of research [149]. Ethical considerations in cancer genomics extend to equity in accessing genetic information and services, the use of genetic information in non-health-related contexts, and the prevention of genetic discrimination [150]. Research activities pose immediate challenges related to obtaining fully informed consent, particularly when genetic information may be repurposed for purposes beyond the original study. As genetic sequencing technologies advance and become more accessible, the ethical principles and historical context underlying clinical genetics are crucial for guiding practice and helping patients navigate the complex medical-psychosocial landscape [151]. Furthermore, the development of precision medicine driven by NGS, information technology, and artificial intelligence raises ethical questions [152, 153]. Empirical studies have explored these issues from the perspectives of healthcare professionals, researchers, and patients. The ethical implications of cancer genomics are part of a broader conversation about genomics and its societal impact. The affordability and accessibility of WGS are ushering in a new era of genomic research, including genome-wide association studies that identify links between genetic variants and complex behaviors and outcomes. The European Commission has engaged in foresight analysis, providing an overview of the ethical, legal, and social implications of genomics [154]. Additionally, the American College of Physicians has released a position paper focusing on ethical considerations in precision medicine and genetic testing within general internal medicine. The ethical and social considerations of cancer genomics are intricate and require careful thought to ensure the protection of individual rights and welfare, the just conduct of research, and equitable access to genetic information and services. These considerations extend beyond healthcare, influencing broader societal and ethical landscapes, necessitating ongoing discussions and policy development to address emerging issues in the ever-evolving realm of genomic healthcare [155].

## 8 FUTURE DIRECTIONS

The rapid progression of cancer genomics in recent years has significantly influenced clinical cancer care. Deep sequencing has unraveled the complexities of clonal heterogeneity, shedding light on acquired resistance mechanisms. The concept of blood-based monitoring through liquid biopsy has emerged as a sensitive and cost-effective proxy for tumor response, addressing challenges in obtaining recurrent tumor biopsy materials. Immunogenomics, driven by genomic studies, has transformed the landscape of cancer immunotherapy. The correlation between high mutational loads and positive responses to checkpoint blockade therapy has challenged traditional definitions of actionable mutations. The potential use of immunotherapies as a second-line approach following DNA-damaging chemotherapy opens new avenues for treatment.

Genomics-driven efforts to identify tumor-specific neoantigens for personalized vaccine development showcase the potential of the field. Despite the progress, cancer remains a formidable challenge, and ongoing clinical and translational efforts in cancer genomics promise continued impact. The interplay between genomics, liquid biopsy, and immunogenomics holds the key to advancing personalized and effective cancer treatments. As research unfolds, the results are anticipated with excitement, offering hope for further breakthroughs in cancer medicine.

## CONCLUSION

The evolution of cancer genomics, rooted in historical discoveries, has led to significant advancements in understanding and treating cancer. Genomic technologies, liquid biopsy, immunogenomics, ML, and targeted therapies have reshaped cancer research and care. Challenges such as intratumor heterogeneity persist, but promising developments, including combination therapies, are emerging. Ethical considerations in genetic information use and the societal impact of genomics require ongoing attention. As we navigate this dynamic landscape, interdisciplinary collaboration and ethical reflection will be crucial for realizing the full potential of genomic healthcare in cancer.

## Figures and Tables

**Table 1:** Clinical trials with their NCT number of genomic studies of cancer.

**Clinical Trial**	**NCT Number**	**Phase**	**Outcome**	**References**
Prostate cancer genomic heterogeneity (PROGENY)	NCT02022371	2	The trial identified key genomic alterations driving tumor heterogeneity, clonal evolution, and metastasis. Plasma DNA sequencing and imaging provided valuable biomarkers for drug resistance and therapeutic response in prostate cancer, as well as for tracking disease progression.	[13]
Deciphering antitumor response and resistance with intra-tumor heterogeneity (DARWINII)	NCT02314481	2	The trial provided insights into progression-free survival, objective response rates, and overall survival. It identified molecular resistance mechanisms, tracked tumor evolution *via* circulating tumor DNA [ctDNA], and assessed treatment toxicity, aiding personalized prostate cancer management.	[14]
OFS in premenopausal node + breast cancer with low genomic risk (INTERSTELLAR)	NCT05333328	4	The study evaluated distant recurrence-free and invasive disease-free survival over five years. The findings highlighted key factors influencing recurrence and progression of invasive disease, offering valuable prognostic insights to improve post-surgical cancer management and long-term patient outcomes.	[15]
Study of Trastuzumab Deruxtecan [T-DXd] *vs* Investigator's Choice Chemotherapy in HER2-low, Hormone Receptor Positive, Metastatic Breast Cancer (DB-06)	NCT04494425	3	The trial assessed progression-free survival, overall survival, objective response rate, and duration of response in patients with HR+, HER2-low breast cancer. Trastuzumab deruxtecan demonstrated clinical benefit, characterized by improved disease control, manageable safety, and preservation of quality of life over 60 months.	[16]
CFI-400945 in patients with advanced/metastatic breast cancer	NCT03624543	2	The trial evaluated the CFI-400945 disease control rate, safety, and molecular effects in tumors over a two-year period. It identified potential biomarkers of response through Next-Generation Sequencing (NGS) and immunohistochemistry, with manageable adverse events and promising molecular alterations linked to treatment efficacy.	[17]
Adjuvant PD-1 Blockade for High-risk Stage-II DMMR/​MSI-H Colorectal Cancer (SMALLER)	NCT06520683	3	The trial assessed disease-free and overall survival over a three- to five-year period, comparing treatment arms using log-rank tests. Adverse events were evaluated, revealing manageable safety profiles with statistically significant comparisons of immune-related and overall adverse event rates.	[18]

**Table 2:** Intra-tumor heterogeneity and the genomically altered genes across different cancer types, as indicated by TCGA data.

**Type of Cancer**	**Types of Heterogeneity**	**Altered Genes**	**References**
Renal	Mutations, chromosome aberrations, and DNA ploidy	*HIF1A, VHL, PBRM1, BAP1, SETD2, MDM4, JAK2, MYC*	[26, 27]
Brain	The mutations, amplification, and expressions of genes associated with oncogenic signaling, cell proliferation, immune response, and hypoxia, along with CD133 expression	*ERBB2, NF1, AKT3, IRS2, PTPRD, MSH2, MSH6, MLHI, TP53, PARK2, FGFR2*	[28-30]
Breast	Genotypic alterations and expressions of cell surface markers	*GATA3, RB1, MAP3K1, PTEN, AKT1, CDH1, MLL3, PIK3CA, TP53*	[31-33]
Colorectal	Expressions of CD133 and receptor tyrosine kinases, along with phosphorylation of signaling proteins	*FBXW7, APC, PIK3CA, KRAS, TP53, CTNNBI, SMAD2, IGF2, FAM123B, SMAD4, TCF7L2, NRAS*	[34, 35]
Lung	DNA ploidy, mutations in EGFR and BRAF V600E, expressions of p53, c-Myc, and Ki-67, along with phosphorylations of signaling proteins.	*EGFR, KRAS, MDM2, TERT, NKX21, HRAS, NRAS, MAP2K1, ERBB2, RBM10, RITI, NF1, TP53*	[36, 37]
Head and neck	Genotypic alterations	*SOX2, TP63, NFE2L2, FAT1, AJUBA, NOTCH1, CASP8, HRAS, CDKN2A, E2F1, TRAF3, PIK3CA*	[38, 39]

**Table 3:** Comparison of different liquid biopsy markers, including CTCs, ctDNA, and exosomes.

**From**	**CTCs**	**Exosome**	**ctDNA**
Stability	High	High (protection by phospholipid bilayer)	Relatively low (short half-life)
Source	Blood, Urine, Cerebrospinal fluid, *etc*.	Urine, blood, ascites, cerebrospinal fluid, pleural fluid, *etc*.	Urine, blood, saliva, cerebrospinal fluid, synovial fluid, *etc*.
Information load	Complete genetic information, including genome, transcriptome, and epigenetic variation	Carry RNA, proteins, and other biomolecules	Carry information on multiple genetic variants
Scale	Urine, blood, cerebrospinal fluid, *etc*.	Urine, blood, ascites, cerebrospinal fluid, *etc*.	Nanoscale (DNA fragments)
Heterogeneity	High (large variations)	Moderate	Low
Difficulty of standardization	High	High	Moderate
Rarity	High	Moderate (interference from other vesicles in body fluids)	High (in early-stage tumors)
Technical difficulty	High (complex enrichment, identification techniques)	Medium (depends on detection techniques)	Moderate (relies on high-sensitivity detection technology)
Clinical significance	Drug resistance mechanism studies, prognostic assessment, and drug sensitivity prediction	Drug response monitoring, early diagnosis, prognostic assessment	Minimal Residual Disease (MRD) testing, early screening
Operating difficulty	High (multi-step process)	Moderate	Low

**Table 4:** Immunogenomics incorporated cancer treatment.

**Treatment Modality**	**Description**	**References**
Cancer Vaccines	Immunogenomics technologies correlate genetic abnormalities with anti-tumor immunity, enabling the design of tumor vaccines.	[94]
Targeted Therapy and Immunotherapy	Advances in NGS enhance cancer genomic research, leading to a better understanding of antitumor immunity. Personalized immunotherapies target highly cancer-specific neoantigens derived from somatic mutations.	[95]
Checkpoint Inhibitors	Technological advances in cancer immunity, including immune checkpoint blockades, have been successful in cancer treatment. Immunogenomics helps in evaluating the immune status of tumors and predicting treatment responses using computational methods.	[96]
Chimeric Antigen Receptor T-cell (CAR-T cell) Therapy	Immunogenomics has the potential to enhance personalized cancer treatment by integrating conventional approaches with immunotherapy, such as surgery, chemotherapy, and radiation therapy. CAR-T cell therapy is emerging as a promising option in cancer treatment.	[97]

**Table 5:** Current genomic revolutionary approaches.

**Focus**	**Key Finding**	**References**
Evaluate the concordance and utility of liquid biopsy (specifically, ctDNA) compared to standard tissue-based methods in identifying actionable genomic alterations in NSCLC patients.	ctDNA-based NGS testing significantly shows actionable genomic biomarkers for treatment selection	[141]
Neoantigens in the context of glioblastoma multiforme (GBM), research for targeted therapy (major histocompatibility complex (MHC) class I-restricted KRAS^G12D^ mutation and an MHC class II-restricted ERBB2IP^E805G^ mutation) and immunotherapy.	The identification of neoantigens specific to an individual patient's tumor holds significant promise for the development of personalized immunotherapies.	[142]
CRISPR/Cas9 technology and CAR-T cell therapy in the context of cancer immunotherapy.	CRISPR/Cas9 technology, as a genome engineering tool, has improved the safety of CAR-T cells for cancer immunotherapy.	[143]
Employ comprehensive WGS for personalized Breast Cancer treatment.	Enable precision medicine and reduce adverse drug reactions.	[144]
Chemical modifications to DNA and proteins that do not change what the gene encodes	Highlight its viability for use in clinical trials and eventual integration into early cancer detection practices.	[145]
Prophylactic therapy, called a biological modifier to *Breast Cancer Gene 1 (BRCA)* and *BRCA2* genes, is susceptible to breast and ovarian cancer.	Reduce chronic inflammation or metabolic syndrome that can contribute to cancer growth.	[146]

## LIST OF ABBREVIATIONS

AI: Artificial Intelligence
ARMS: Amplified Refractory Mutation System
BRCA: Breast Cancer Gene
CAR-T Cell: Chimeric Antigen Receptor T cell
Cas9: CRISPR Associated Protein 9
CDK: Cyclin-Dependent Kinases
cfDNA: Circulating Free DNA
CRISPR: Clustered Regularly Interspaced Short Palindromic Repeats
CTCs: Circulating Tumor Cells
ctDNA: Circulating Tumor DNA
CTLA-4: Cytotoxic T Lymphocyte Antigen 4
ddPCR: Droplet Digital PCR
EGFR: Epidermal Growth Factor Receptor
GBM: Glioblastoma Multiforme
HCC: Hepatocellular Carcinoma
HER2: Human Epidermal Growth Factor Receptor 2
ICGC: International Cancer Genome Consortium
ICIs: Immune Checkpoint Inhibitors
IHC/FISH: Immunohistochemical/Fluorescence *In Situ* Hybridization
KRAS: Kirsten Rat Sarcoma Virus
MHC: Major Histocompatibility Complex
ML: Machine Learning
MPS: Massively Parallel Sequencing
MRD: Minimal Residual Disease
MSI: Microsatellite Instability
NGS: Next-Generation Sequencing
NSCLC: Non-Small Cell Lung Cancer
PCR: Polymerase Chain Reaction
PD-1: Programmed Death 1
PD-L1: Programmed Death-Ligand 1
SARS-CoV-2: Severe Acute Respiratory Syndrome Coronavirus 2
SEPT9: Septin-9
TCGA: The Cancer Genome Atlas
TEVs: Tumor Extracellular Vesicles
TMB: Tumor Mutational Burden
TP53: Tumor 53
WES: Whole-Exome Sequencing
WGS: Whole-Genome Sequencing
WGTS: Whole-Genome Transcriptome Sequencing
WTS: Whole-Transcriptome Sequencing

## DECLARATION

The English language of the article was improved with ChatGPT. Upon generating draft language, the author reviewed, edited, and revised the language to their liking and takes ultimate responsibility for the content of this publication. During the preparation of this work, the authors utilized the GPT-4 model of ChatGPT (OpenAI’s large-scale language-generation model) to enhance the language of the article.
